# The age of abundant scholarly information and its synthesis– A time when ‘just google it’ is no longer enough

**DOI:** 10.1002/jrsm.1520

**Published:** 2021-09-07

**Authors:** Michael Gusenbauer

**Affiliations:** ^1^ Department of Strategic Management, Marketing and Tourism University of Innsbruck Innsbruck Austria; ^2^ Chair of Strategy and Organization, Technical University of Munich Munich Germany

**Keywords:** abundance of scholarly information, conduct and reporting guidance, evidence synthesis, librarians, search literacy, systematic reviews and meta‐analyses

## Abstract

Academic research has changed in recent years. It has entered the age of abundant scholarly information. New scientometric data shows impressive increases in both the quantity and quality of information researchers produce. Since 2007 about the same number of publications have become accessible on databases as more than the hundred years prior. At the same time, evidence synthesis has become key in making this wealth of information understandable and useful. Researchers need to be increasingly proficient in identifying relevant information – to be able to build on an increasingly comprehensive research base and to adhere to rising standards in evidence synthesis. Both these requirements make a ‘true partnership between librarians and researchers’ in demand like never before.

**Highlights**

**What is already known?**

- Today we researchers feel the benefits of abundant scholarly information, but also the downsides of information overload.
- How well researchers are able to access relevant information is increasingly a determinant for the quality of their work, particularly for conducting rigorous evidence synthesis.

**What is new?**

- Scientometric analysis shows how systematic reviews and meta‐analyses have risen – from their humble beginnings in the 1970s – to accounting for up to 1.79% of all published studies in 2020.
- The quality of evidence synthesis is increasing: studies increasingly cite conduct and reporting guidance, introducing higher levels of rigour and accountability.

**Potential impact for RSM readers outside the authors' field?**

- Evidence synthesis has gained importance in all science disciplines – not only in the health sciences but particularly also in the life and social sciences.
- Librarians can broaden their reach by targeting disciplines that increasingly adopt evidence synthesis, yet lack behind in (adequately) using conduct and reporting guidance.

## 1 INTRODUCTION

I am happy our commentary^1^ has drawn the attention of Cornell University librarians who point out ways in which librarians support researchers engaging in lookup, exploratory or systematic searches.^2^ I believe this dialogue is a great vehicle to raise awareness of the importance of information literacy and make readers aware of resources available at their institutions and beyond. My response seconds the authors' call for a ‘true partnership between researchers and librarians’ and puts this relationship into the perspective of a rapidly changing (re)search environment: the age of abundant scholarly information and evidence synthesis. With new scientometric data, this commentary illustrates the new realities in which researchers work and how the challenges associated with information abundance have changed searching over the last years (methodology see Data S1). In my view, the expertise of librarians can be of particular value if these new realities are factored in when approaching information seekers that require help – knowingly or unknowingly.

## 2 INFORMATION ABUNDANCE AND THE RISE OF META‐ANALYSES AND SYSTEMATIC REVIEWS

The *value* of the supply of scholarly information is foremost determined by how effectively the ones that seek information are able to find what they need. When they find, the supply of scholarly information gets valuable: with relevant evidence and research findings at hand, learning takes place, and better decisions are made. I claim, the demand side of scholarly information – that is, the abilities and skills to best access scholarly information – becomes increasingly important. With rising standards of what constitutes rigorous (re)search practise, researchers need to, more than ever, know how to find effectively amongst the rapidly growing wealth of information.

### 2.1 New *quantities* of information supply

**FIGURE 1:**
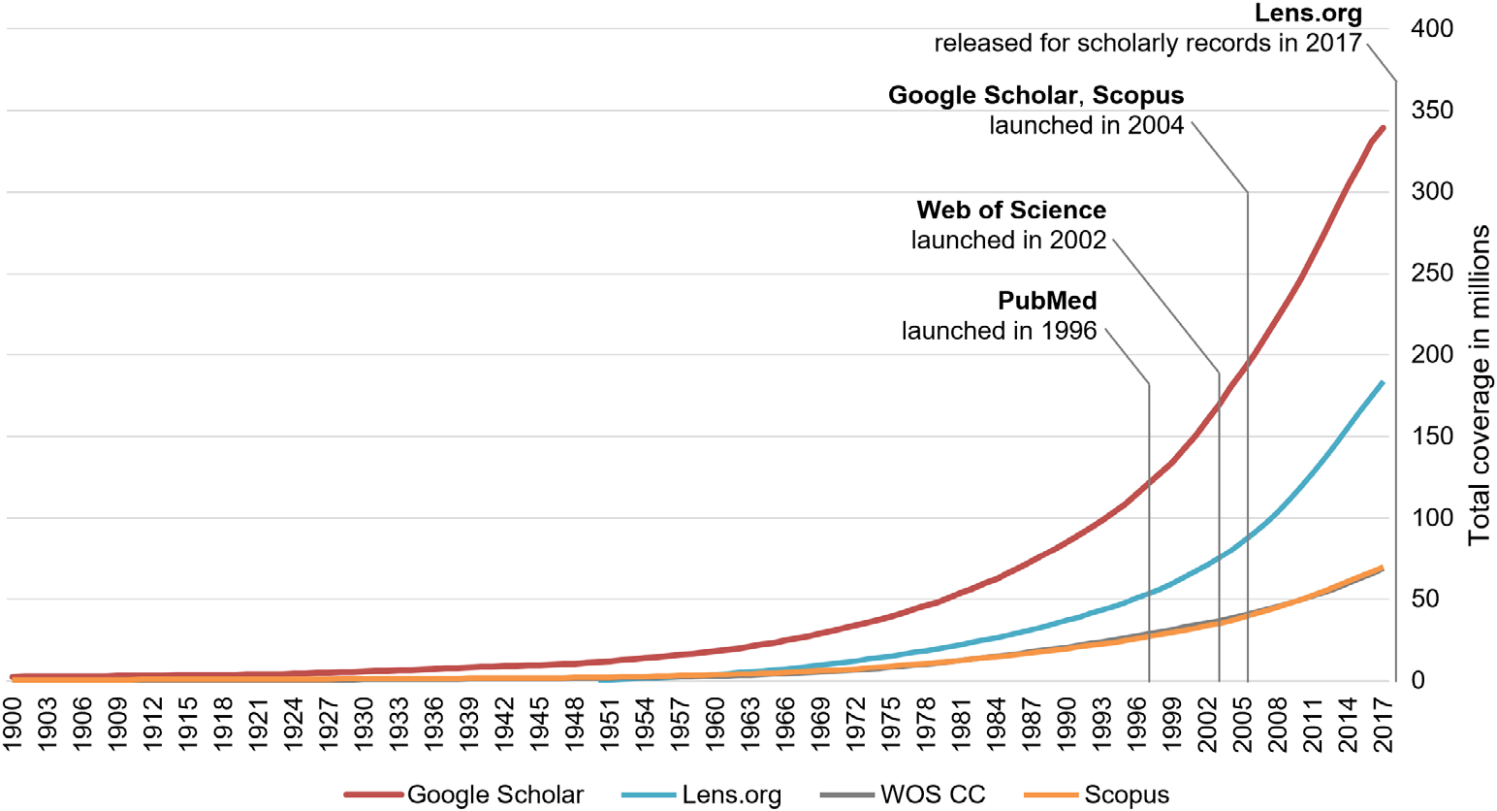
Exponential growth of scholarly records accessible at major search systems (between 1900 and 2017). Large differences in retrospective coverage are visible between curation‐based systems (WOS Core Collection [CC], Scopus) and systems that include crawlers (Google Scholar, Lens.org). The slight drop in yearly coverage of Google Scholar (after 2013) and Lens.org (after 2016) is attributable to lagging crawler‐based indexing of scholarly records. WOS CC and Scopus as curation‐based systems do not show these lags. Methodology and exact WOS CC indexes see Data S1 [Colour figure can be viewed at wileyonlinelibrary.com]

The supply of information available to researchers has increased exponentially in the last decades. In 1982 a researcher might have still gotten away with the statement: ‘I know all important people in my discipline, I do not need databases’^3^ ^(p2)^. At the time of the statement, this person had a total of 13.7 million scholarly records at hand (see Figure 1).* Within the next 15 years, science produced the same amount as in the more than a hundred years prior. At present, the publication of scientific records has sped up to the point that researchers produce these 13.7 million records in just 4 years. Innovations in academic searching such as PubMed (1996), Web of Science (2002), Google Scholar (2004) or Scopus (2004) were a reaction to suboptimal information discovery prior. Particularly search systems that (partially) rely on data obtained via crawlers, such as Google Scholar^4^ or recently released Lens.org (2017), have allowed access to a large amount of Grey literature, not indexed by established citation indexes (such as the Science Citation Index). Figure 1 highlights the phenomenal number of scholarly records made available by these crawler‐based systems in comparison to Scopus or the Web of Science Core Collection (WOS CC).

### 2.2 New *qualities* of information supply

**FIGURE 2:**
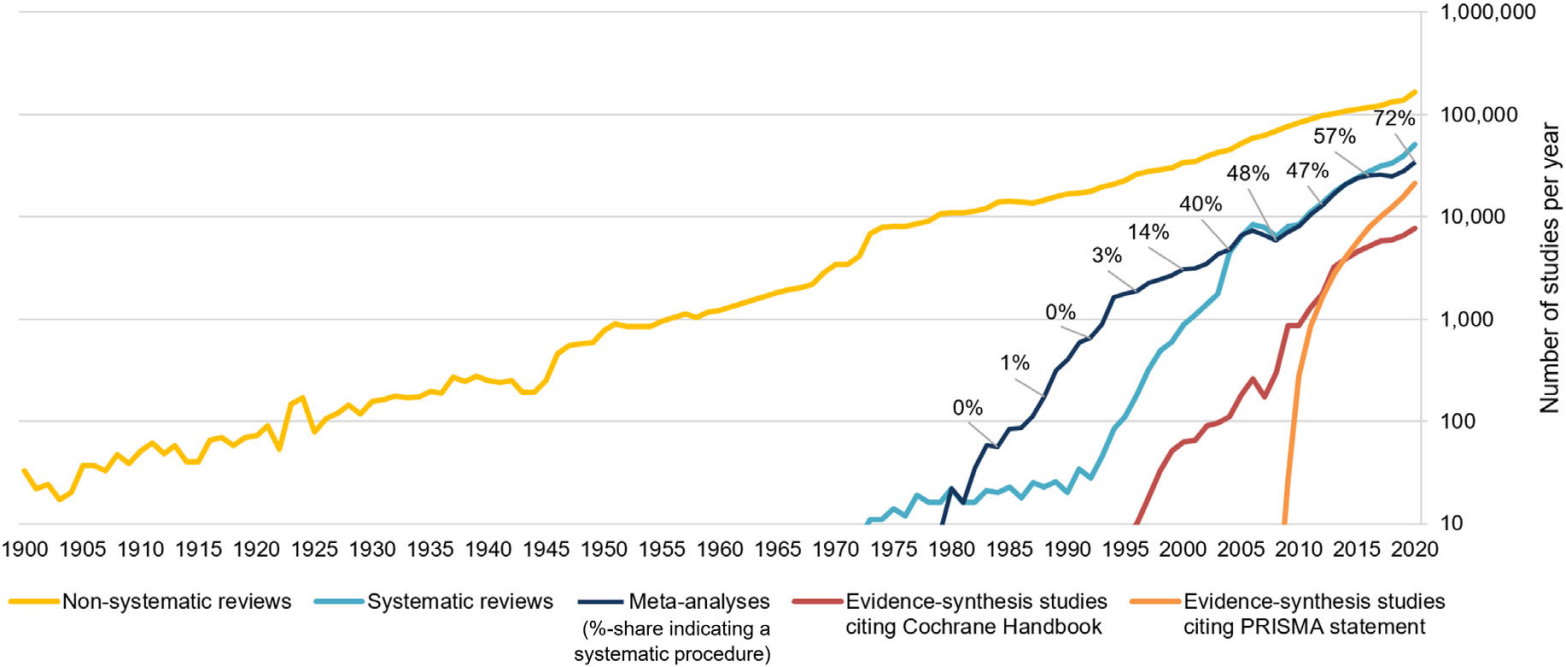
The rise of systematic reviews and meta‐analyses since the 1970s (1900–2020, logged scale). Yearly publication estimates of different review types (non‐systematic reviews, meta‐analyses and systematic reviews) and review guidance (Cochrane Handbook, PRISMA statement). Estimation via query hit counts. Methodology see Data S1 [Colour figure can be viewed at wileyonlinelibrary.com]

Next to the *quantity* of information available, also its *quality* improved: with the introduction of evidence synthesis, findings of higher grade and less bias became available. Evidence synthesis lowered the burden of individual information seekers to synthesise the increasing quantity of information themselves. While reviews of all sorts (including traditional/narrative reviews) have appeared since the turn of the 20th century, they became more frequent only after the Second world war (see Figure 2). The 1970s – a time when reviews still became increasingly popular – also experienced the great innovation of *systematic* literature reviews and meta‐analyses. The term meta‐analysis for example was introduced by Glass^5^ ^(p3)^ in 1976 (for a chronology see Gurevitch et al^6^). Since then, the share of meta‐analyses and systematic reviews continuously rose to a level that more than every fourth reviews now states being systematic.† In 2020 alone, scholars published more than 60,000 meta‐analyses and systematic reviews in reputable outlets – in 2008 this number was still at about 10,000 a year.

**FIGURE 3:**
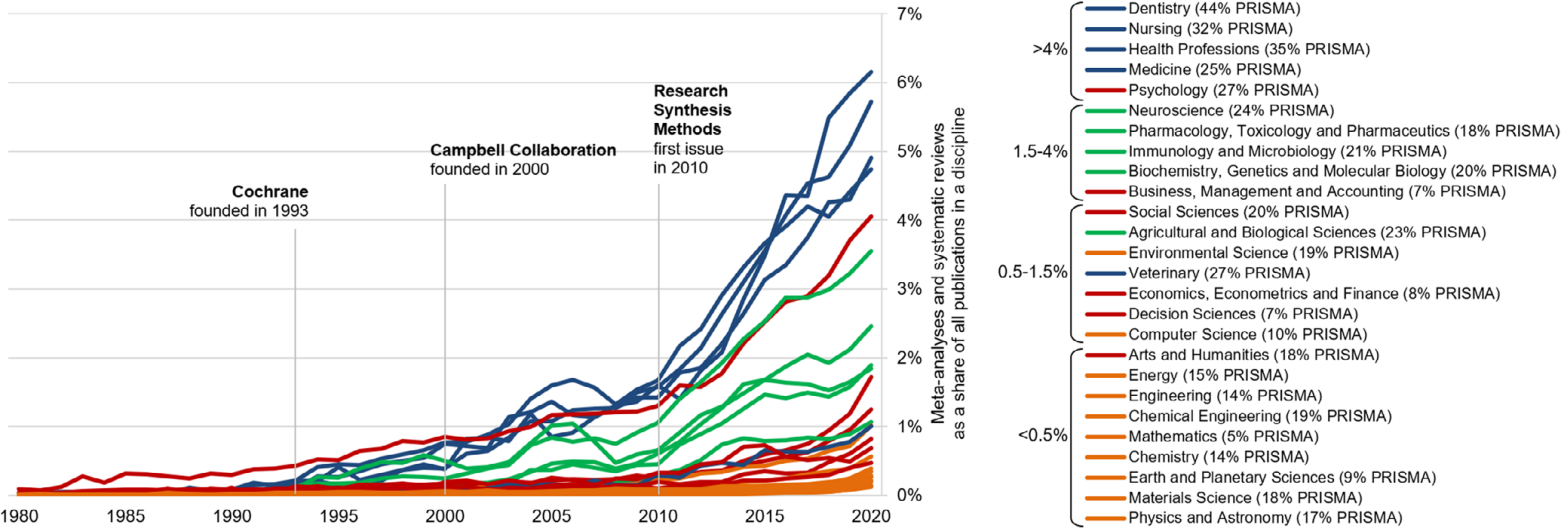
Relevancy differences of evidence synthesis amongst academic disciplines (1980–2020; PRISMA statement citation rate in brackets on right). Historically, evidence synthesis has been most important in health sciences (dentistry; nursing; health professions and medicine). In the 1980s and 1990s however, psychology has been pioneering the methodology and applies it heavily ever since. Life sciences (neuroscience; pharmacology, toxicology and pharmaceutics; immunology and microbiology and biochemistry, genetics and molecular biology) has been the second‐most active field in evidence synthesis. For large parts of physical sciences evidence synthesis is not (yet) relevant – even though there has been some noticeable increase in prevalence since 2015. A study was counted as a meta‐analysis/systematic review if it uses the terms in title, abstract or keywords (see methodology in Data S1). The legend was ranked according to values from 2020; Disciplinary categorisation after All Science Journal Classification used by Scopus with 26 major fields, excluding the multidisciplinary field; Colour code: blue = health sciences, green = life sciences, red = social sciences and orange = physical sciences [Colour figure can be viewed at wileyonlinelibrary.com]

Scopus data shows us the great rise of evidence synthesis in general, and it is varying relative disciplinary significance in specific: after a continuous increase since the 1970s, meta‐analyses and systematic reviews in 2020 accounted for up to 1.79%‡ of all studies published. This rise of evidence synthesis is clearly attributable to the field of health sciences (see Figure 3). Almost 65% of evidence‐synthesis studies were published there. While the relevancy of evidence synthesis varies significantly amongst disciplines, evidence synthesis has become more relevant in every(!) discipline in 2020 compared to 2019 – a trend that has been noticeable in the last years. Particularly since 2008, almost all disciplines have sharply increased the adoption of evidence‐synthesis methods relative to all studies published – a trend illustrated in Figure 3.

With time, not only *traditional* literature reviews were increasingly replaced by *systematic* literature reviews, but meta‐analyses have become substantially *more systematic* too. Until the millennium meta‐analyses were largely not referring to terms that would indicate ‘systematic’ procedures. This has considerably changed to the point that in 2020, 72% of all meta‐analyses refer to some kind of systematic approach – either in searching, conducting or reporting. Continuously increasing adoption rates of PRISMA^7^ reporting guidance amongst meta‐analyses substantiate increasingly systematic approaches amongst meta‐analyses. The trend towards systematic approaches received traction by the formation of organisations dedicated towards evidence‐based practises, such as Cochrane (in 1993) and the Campbell Collaboration (in 2000). Together with methodological journals such as Research Synthesis Methods, it has been the guidelines from these and other organisations that promoted more rigorous reporting and conduction of meta‐analyses (e.g., PRISMA statement^7^ or Cochrane Handbook^8^).

While the spread of the systematic review and meta‐analysis methods provide an ever‐increasing supply of high‐quality information, there is also a caveat. Not everything that calls itself a meta‐analysis is free from bias and not everything that states being systematic, are in fact systematic.^9^ John Ioannidis, a co‐author of the PRISMA statement, describes how for example ‘meta‐analyses are emerging as a powerful marketing tool’^10^ ^(p495)^ – a statement pointing to authors' preference for publication success and high citation rates,^11^ rather than for the most rigorous research designs. Thus, while quantitative data indicates an increase in the quality of information supply – that is, more evidence‐syntheses with overall more rigour in reporting and conduct – the quality of individual studies varies however.^12^

## 3 INFORMATION DEMAND: AN INDIVIDUAL SKILL

After we looked at the significant improvements in the quantity and quality of information supply, we also need to look at the demand side – the skills and practises of information seekers that influence the quality of research. The dominant way of searching for information online – that is, using Google with a continuous global market share of above 90%^13^ – has trained us researchers to equate searching with ‘googling’. Cornell university librarians point to this issue: ‘Busy researchers will often discount effective search methodology, relying on their daily experience 'with internet search engines’.^2^ We researchers are used to impatiently collecting information with the idea that what ranks high^14^ is perceived most relevant. A ready answer is right at our fingertips and on top of the search engine results page.^15^ Yet, I claim that this googling mentality is only adequate for lookup searching,^16^ the type of searching Google and Google Scholar are predominantly geared towards. For exploratory and systematic searching, however, fast contentment with high‐ranking results might be problematic as it reduces the quality of search outcomes.

### 3.1 Satisficing behaviour

Fast contentment occurs as soon as the first option seems adequate – a behaviour also understood as *satisficing*.^17^ Satisficing means searching a haystack for ‘[the first] needle sufficiently sharp enough to sew with’. *Optimisation* rationale runs counter to satisficing where an individual seeks out the best possible solution. An optimiser would search for ‘the sharpest needle in a haystack’^18^ ^(p11934)^ or in the case of systematic searching, *for all sharp needles in a haystack*. Satisficing behaviour is generally rational,^19^ as increasingly long searches might mean decreasing marginal rates of return and increasing opportunity costs^18^ ^(p11934)^. Yet, it is particularly difficult to estimate these costs, and in practise, searchers often stop when first reaching the perceived requirements. Interpretation of when requirements are met will depend on the level of ambition of a searcher – which is sometimes guided by what one thinks one gets away with.

In the case of systematic searches, the introduction of conduct guidance – with the prominent example of the ‘Cochrane Handbook’^8^ – has narrowed the scope for subjective interpretation of search requirements. In systematic reviews, the general goal is to identify *all* relevant records that meet the eligibility criteria.^8^, ^20^, ^21^ Yet, given the substantial time constraints of exhaustive searches, some guidance has explicitly weakened the exhaustiveness criterion by accounting for the ‘resource constraints’ of searchers.^20^ The introduction of rapid reviews as a somewhat watered‐down version of systematic reviews has addressed the same issue, favouring completion time over exhaustiveness. Both these attempts aim at reducing the escalating opportunity cost of perfectly exhaustive searches^22^ weighting the satisficing rationale with the optimisation rationale; a logic that makes particular sense when evidence‐based decisions need to be made fast.

### 3.2 Guidance

The scientometric analysis found that these reporting and conduct guidance have been increasingly adopted over time (see Figure 2). In 2020 already about 39% of systematic reviews and meta‐analyses referred to either the PRISMA statement or the Cochrane Handbook. Particularly the PRISMA statement has been increasingly cited – in 2020 already more than a third of systematic reviews and meta‐analyses. Figure 3 shows the citation rates of PRISMA guidance vary substantially across disciplines, with dentistry (44%) and health professions (35%) having the highest, and mathematics (5%), business, management and accounting (7%), and decision sciences (7%) the lowest rates. Increased citation of guidance is positive as it signals researchers' awareness for methodological rigour (compared to studies that omit guidance) and it brings new levels of accountability to these studies compared to unguided approaches. Conversely, low citation rates are likely to hint at a higher need to improve methodological rigour of evidence synthesis in these disciplines. It needs however to be remembered that in too many cases the *citing* of guidance does not mean it was applied appropriately.^9^, ^23^ Thus, while higher citation rates of reporting or conduct guidance signal increased awareness for methodological rigour, it is also important to make sure guidance is properly applied.

Closely associated with these new levels of rigour and accountability, the analysis shows that more and more systematic reviews and meta‐analyses report the systems they searched with. Over time, a considerably larger share of studies reported a selection of 14 popular systems that are particularly suitable for systematic searching.^24^ Upon randomly reviewing evidence‐synthesis articles, I however also found that still, many researchers do not state the exact (combination of) search systems and databases they searched with. This lack of reporting, unfortunately, reduces transparency and limits reproducibility of search strategies (common practise is for example to only mention the Medline database, not referring to whether it was accessed via PubMed, ProQuest, Web of Science, Ovid or some other system).

### 3.3 Inadequate systems use

Another area with room for improvement is education on which systems are suitable for specific search types. My analysis of 16,732 systematic reviews and (systematic) meta‐analyses shows an increasing number include Google Scholar in their searches for evidence – In 2017 this number was already at 15% (see Table 1). While searching multiple systems is generally advisable,^20^ it can be detrimental if systems are used the wrong way. Randomly screening the full texts of evidence‐synthesis studies that used Google Scholar, I found that many used the system for Boolean searches, a heuristic the system is technically unsuitable for. Our previous study^24^ documented these flaws in Google Scholar's service which persist to this day. The problems with Boolean searches do however not mean that Google Scholar cannot be used as a supplementary system in evidence synthesis – for example for citation chasing of Grey literature. The problem is that many of the 15% of the meta‐analyses and systematic reviews that used Google Scholar, used it like they would PubMed, ProQuest, Scopus or Web of Science. These issues need to be communicated or else a fraction of evidence synthesis will continue being biased and irreproducible.

**TABLE 1:** Key findings on searching and reporting practises in evidence‐synthesis studies [Colour table can be viewed at wileyonlinelibrary.com]

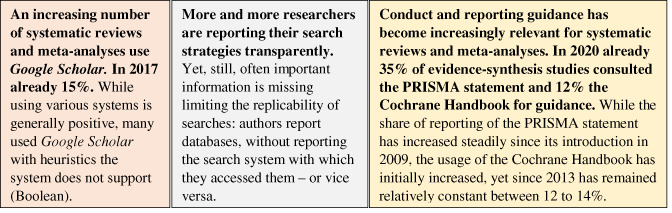

*Notes*: Box 1 and 2: Findings from Lens.org searching the full texts of 16,732 systematic reviews and meta‐analyses (without subject limit, yet most identified systematic reviews/meta‐analyses were published in various health sciences); Box 3: Findings from Scopus search.

## 4 RECOMMENDATIONS TO IMPROVE SEARCH OUTCOMES

Despite the general trend towards higher quality in information supply, more rigour could be achieved through more thoroughly adopted guidance on what constitutes good searching. To improve search outcomes, we need to place ourselves in the position of the information seeker. For many information seekers, the threshold for an ‘acceptable result’ is exactly the point where an external evaluator (if existent) deems it satisfactory. Students stop when reaching the minimum number of sources/pages/words for an assignment. Faculty stops when reaching a level of quality, they deem publishable.^25^ As the data shows, conduct and reporting guidance has become increasingly prevalent, indicating that requirements for effective search strategies have become more relevant too. Librarians are important voices to promote the further adoption and proper application of quality standards which again will create demand for their search expertise.

The authors note ‘For many, learning to search is necessary but not compelling unless they are frustrated with information retrieval.’^2^ I claim frustration might also prove beneficial in this context, as it means insufficiencies of the search process are being realised. Frustration significantly motivates search moves and improvements of search outcomes by rationally satisficing individuals.^26^ Frustration most likely comes from one of either two places: first, when searchers were first satisfied, yet then some external authority rated the quality of the search strategy inadequate; or second, searchers themselves realise that search results are inadequate. While in the former case the external authority guides improvement, searchers need help to adequately interpret frustration in the latter case. Ideally, search support would help searchers during the search task, either through personal guidance (relatively costly) or automated support via online tools. Generally, I think it is important to elevate the satisficing level of searchers in search education. In times of increasing information abundance, inadequate search results are also increasingly often the result of inadequate searching skills and not due to the lack of information available. Another way of elevating the satisficing level is by more frequent use of external authorities that provide guidance on when searching has been good enough (e.g., Cochrane Handbook). Detailed guidance on systematic searching already exists, yet has not become common knowledge amongst editors and reviewers, not to speak of authors. When information seekers learn about the threshold for acceptable results is in fact higher, many will have an incentive to improving their search strategy.

A promising way forward might also be the introduction of *exploratory search guidance* similar to existing guidance for systematic searches. As the goal of exploratory searching – understanding a previously relatively unknown topic – is inherently vague, this guidance cannot be as concrete as the one for systematic searching. Nevertheless, the introduction of stopping rules might prove helpful for *scoping or mapping studies* ^27^ – in particular to avoid cases where weak search skills and not the unavailability of records determine a dissatisfactory search outcome. In cases with dissatisfactory results a searcher might be instructed to first perform these three steps to improve exploration (based on the ‘search triangle’^1^): first, searching alternative search systems. Second, searching with alternative terms (synonyms, antonyms, hyponyms, hypernyms). Third, searching with different heuristics (Boolean, citation chasing, filtering, controlled vocabularies) – also via new (exploration) tools that become increasingly powerful (e.g., Citation Chaser, Litmaps or Connected Papers). Additionally, using new sorting options of search results allows the surfacing of articles based on innovative metrics – for example, Dimensions.ai has introduced sorting based on field citations or the Altmetric Attention Score or Web of Science offers sorting based on usage data. Another helpful innovation for exploratory searching might be text searches, where searchers upload abstracts or text parts as seed input to identify results (e.g., Dimensions.ai Abstract search, Elsevier JournalFinder or JSTOR Text Analyser). These ideas could be first steps to discuss the improvement of exploratory search phases which are important determinants of the quality of spotting research gaps, developing research designs or the theoretical positioning of studies.

## 5 CONCLUSION

Librarians' expertise is in demand like never before. The data illustrates that this demand comes from five places: first, an exponentially increasing number of scholars engage in more complex searches. Compared to 2008, in 2020 about six times more evidence‐synthesis studies were published. These syntheses require both effective exploratory searching skills (scoping) and systematic searching skills. Second, these searchers need to weed through an exponentially increasing number of scholarly records. This year, researchers need to search through double the studies compared to researchers in 2007. Third, conduct and reporting guidance become increasingly important. Compared to 2017, now almost double(!) the number of studies refer to the Cochrane Handbook or the PRISMA statement (still too many do not use it adequately though). Fourth, searchers need to be educated in an increasing number and rapidly updating search and discovery tools. Lens.org, Meta.org or Dimensions.ai are only some examples of the dozens of systems and tools that were introduced to improve finding in the last years. Fifth, evidence syntheses became popular in non‐health‐related fields. The analysis shows a steep increase in social sciences and life sciences in the last few years. The fact that PRISMA guidance is not evenly adopted amongst disciplines might be a helpful hint for librarians to draw attention to disciplines that both increasingly rely on evidence synthesis, and have not yet realised the intricacies of information identification. In general, the data presented in this commentary shows the advancement of science, but also that information literacy, more than ever, is an essential skill in academia. I hope these arguments can be useful to promote the ‘true partnership between researchers and librarians’ – for more valuable scholarly information and less frustrated researchers.

## Supporting information


**Data S1.** Supporting information.

## Data Availability

The data that support the findings of this study are available from the corresponding author upon reasonable request. The detailed methods with which the data was obtained can be found in the Data S1. Interested readers can download, replicate and verify the data themselves via the search strategies described there.

## Endnotes

* Estimation based on current coverage of Scopus from 1866–1982. The records available in 1982 might have been even fewer due to weaker search infrastructure and indexing coverage.

† Estimation of the prevalence of review studies is based on counting the studies referring to specific terms is title, abstract, keywords. The exact methodology can be found in the Data S1.

‡ Estimated by counting the mentions of the systematic review method or meta‐analysis in title, abstract or keywords.
