# Supplementary material for: The age of abundant scholarly information and its synthesis– A time when ‘just google it’ is no longer enough
Source: Res Synth Methods. 2021 Sep 7;12(6):684–91. doi: 10.1002/jrsm.1520 (PMC9291810; doi:10.1002/jrsm.1520)
Supplement: Supplementary file 1 — Data S1. Supporting information. [file JRSM-12-684-s001.docx]

# Appendix

**Data collection Figure 1:**

| Google Scholar | *Retrieval date: 24.11.2017*  Entire index  Determined via: coverage data was collected with the methodology described in the 2019 Scientometrics paper on coverage comparison.^[[1]](#footnote-1)^ This methodology is not replicable at the moment due to Google Scholar’s changes in search logic. Similar data was however also obtained by another research group^[[2]](#footnote-2)^) |
| --- | --- |
| Lens.org | *Retrieval date: 16.04.2021*  Entire index (consists of Microsoft Academic, Crossref, PubMed, Core, PubMed Central) |
| Scopus | *Retrieval date: 14.04.2021*  Entire index  Determined via: SUBJAREA(AGRI) OR SUBJAREA(ENVI) OR SUBJAREA(SOCI) OR SUBJAREA(ECON) OR SUBJAREA(EART) OR SUBJAREA(ENGI) OR SUBJAREA(COMP) OR SUBJAREA(MEDI) OR SUBJAREA(ARTS) OR SUBJAREA(BIOC) OR SUBJAREA(BUSI) OR SUBJAREA(ENER) OR SUBJAREA(MATH) OR SUBJAREA(CENG) OR SUBJAREA(CHEM) OR SUBJAREA(PHYS) OR SUBJAREA(MATE) OR SUBJAREA(DECI) OR SUBJAREA(IMMU) OR SUBJAREA(VETE) OR SUBJAREA(NURS) OR SUBJAREA(PHAR) OR SUBJAREA(PSYC) OR SUBJAREA(HEAL) OR SUBJAREA(NEUR) OR SUBJAREA(DENT) |
| Web of Science – Core Collection | *Retrieval date: 16.04.2021*  Science Citation Index Expanded (SCI-EXPANDED) --1900-present  Social Sciences Citation Index (SSCI) --1900-present  Arts & Humanities Citation Index (A&HCI) --1975-present  Conference Proceedings Citation Index- Science (CPCI-S) --1990-present  Conference Proceedings Citation Index- Social Science & Humanities (CPCI-SSH) --1990-present  Book Citation Index– Science (BKCI-S) --2010-present  Book Citation Index– Social Sciences & Humanities (BKCI-SSH) --2010-present  Emerging Sources Citation Index (ESCI) --2015-present  Current Chemical Reactions (CCR-EXPANDED) --1985-present (Includes Institut National de la Propriete Industrielle structure data back to 1840)  Index Chemicus (IC) --1993-present  Determined via: “:” search |

**Data collection Figure 2:**

I used a scientometric methodology to estimate the number of studies via keyword queries. This methodology is for example also used in research evaluation by Elsevier and Times Higher Education to rank universities according to their relative research impact on the UN Sustainable Development Goals^[[3]](#footnote-3)^.

*Why did I use Scopus data and not Lens.org, Google Scholar or Web of Science Core Collection (CC) for most parts of our analysis?*

Lens.org is limited in retrospective coverage to 1950-current (with a substantial lack of coverage of records from the content it deems unknown record types due to its partially use of crawlers). Scopus is the more established, high-quality data provider, relying on human-controlled curation techniques.

Google Scholar does not support our methodology.

Web of Science Core Collection is no unified corpus and the versions I can access have marginally lower coverage than Scopus.

| **#** | **Scope** | **System** | **Search string** |
| --- | --- | --- | --- |
| 1 | Meta-Analyses: TI,AB,KY(MA) | Scopus  (25.04.2021) | TITLE-ABS-KEY({meta analysis} OR {meta-analysis} OR {meta analyses} OR {meta-analyses} OR {metaanalysis} OR {metaanalyses} OR {MASEM} OR {meta analytic} OR {meta-analytic} OR {meta-analytically} OR {metaanalytically} OR {meta-analytical} OR {metaanalytical} OR {metafor} OR {metaSEM} OR {meta-SEM} OR {robumeta} OR {meta regression} OR {meta-regression} OR {meta regressions} OR {meta-regressions} OR {metaregression}) |
| 2 | Systematic Reviews: TI,AB,KY(SLR) | Scopus  (25.04.2021) | TITLE-ABS-KEY({systematic literature review} OR {systematic review} OR {systematic literature reviews} OR {systematic reviews} OR {systematically reviewed} OR {Cochrane review} OR {Cochrane systematic review} OR {Cochrane reviews} OR {Cochrane systematic reviews} OR {Campbell review} OR {Campbell systematic review} OR {Campbell reviews} OR {Campbell systematic reviews} OR {systematicreview} OR {systematicreviews}) |
| 3 | Systematic Meta-Analyses: TI,AB,KY(MA) AND (TI,AB,KY(SLR) OR TI,AB,KY(SS2)) | Scopus  (25.04.2021) | SEARCH STRING #1 AND (TITLE-ABS-KEY({systematic literature review} OR {systematic review} OR {systematic literature reviews} OR {systematic reviews} OR {systematically reviewed} OR {Cochrane review} OR {Cochrane systematic review} OR {Cochrane reviews} OR {Cochrane systematic reviews} OR {Campbell review} OR {Campbell systematic review} OR {Campbell reviews} OR {Campbell systematic reviews} OR {PRISMA statement} OR {systematicreview} OR {systematicreviews} OR {Boolean} OR {Cochrane}) OR ALL({Cochrane Handbook} OR {Cochrane Collaboration Handbook} OR {Cochrane Collaboration Tool Kit} OR {Cochrane Reviewers' Handbook} OR {PRISMA statement} OR {PRISMA 2000 statement} OR {Preferred reporting items} OR {PRISMA-P} OR {PRISMA-E} OR {PRISMA-NMA} OR {PRISMA-Equity} OR {PRISMA-IPD} OR {PRISMA-C} OR {PRISMA-CI} OR {PRISMA-DTA} OR {PRISMA-ScR} OR {PRISMA flow} OR {PRISMA for Abstracts} OR {PRISMA-Children} OR {PRISMA-H})) |
| 4 | All Literature Reviews (NOT: SLR, MA): TI,AB,KY(LR) NOT TI,AB,KY(MA, SLR) | Scopus  (10.06.2021) | TITLE-ABS-KEY({literature review} OR {literature reviews} OR {review of the literature} OR {reviews of the literature} OR {review the literature} OR {reviewed narratively} OR {reviewed the literature} OR {narrative review} OR {narrative reviews} OR {traditional review} OR {traditional reviews} OR {narrativereview} OR {narrativereviews} OR {traditionalreview} OR {traditionalreviews} OR {review study} OR {review studies} OR {review article} OR {review articles} OR {review paper} OR {review papers} OR {integrative review} OR {critical review} OR {critical reviews} OR {comprehensive review} OR {comprehensive reviews} OR {review of} OR {review the} OR {review on} OR {review in} OR {review into} OR {review and} OR {reviewed the} OR {a review} OR {my review} OR {our review} OR {this review} OR {reviewing} OR {reviews of} OR {reviews the} OR {reviews on} OR {reviews in} OR {reviews into} OR {reviewed the} OR {a review}) AND NOT (Search #1 OR Search #2)  *It is difficult to distinguish narrative or traditional literature reviews from other types of reviews, as authors often only refer to ‘review’, rather than specifying the specific type of review they perform. On the contrary, the term review is used differently making estimating the number of review studies according to a specific definition of ‘review’ difficult without manually checking each study. To validate my search string of reviews, I screened the titles, abstracts and keywords of the first 250 records of Google Scholar for ‘review’ and checked whether they would be identifiable with the search string.*  *On the contrary, systematic reviews and meta-analyses more frequently explicitly state their specific labels, making them easier to identify.* |
| 5 | SLR/MA | Scopus  (25.04.2021) | SEARCH STRING #1 OR SEARCH STRING #2 |
| 7 | SLR/MA + Cochrane Handbook | Scopus  (25.04.2021) | SEARCH STRING #5 AND ALL({Cochrane Handbook} OR {Cochrane Collaboration Handbook} OR {Cochrane Collaboration Tool Kit} OR {Cochrane Reviewers’ Handbook})) |
| 8 | SLR/MA + PRISMA | Scopus  (25.04.2021) | SEARCH STRING #5 AND ALL({PRISMA statement} OR {PRISMA 2000 statement} OR {Preferred reporting items} OR {PRISMA-P} OR {PRISMA-E} OR {PRISMA-NMA} OR {PRISMA-Equity} OR {PRISMA-IPD} OR {PRISMA-C} OR {PRISMA-CI} OR {PRISMA-DTA} OR {PRISMA-ScR} OR {PRISMA flow} OR {PRISMA for Abstracts} OR {PRISMA-Children} OR {PRISMA-H})) |

**Data collection Figure 3:**

Scopus data based on SEARCH STRING #5 and subject coverage operators for health sciences, life sciences, physical sciences and social sciences. Retrieved on 28.04.2021.

**Data collection Table 1:**

The full-text search option is provided by Lens.org as one of the very few systems that do that. While Lens.org covers 228 million records, at the time of analysis (16.04.2021) only 1.98 million full texts were searchable and thus analysed to gain a quantitative view on evidence-synthesis search methodology.

For analysing the prevalence of guidance within systematic reviews and meta-analysis I used Scopus data.

| **#** | **Scope** | **System** | **Search string** |
| --- | --- | --- | --- |
| 6 | SLR/MA + Guidance | Scopus  (25.04.2021) | SEARCH STRING #5 AND ALL({Cochrane Handbook} OR {Cochrane Collaboration Handbook} OR {Cochrane Collaboration Tool Kit} OR {Cochrane Reviewers' Handbook} OR {PRISMA statement} OR {PRISMA 2000 statement} OR {Preferred reporting items} OR {PRISMA-P} OR {PRISMA-E} OR {PRISMA-NMA} OR {PRISMA-Equity} OR {PRISMA-IPD} OR {PRISMA-C} OR {PRISMA-CI} OR {PRISMA-DTA} OR {PRISMA-ScR} OR {PRISMA flow} OR {PRISMA for Abstracts} OR {PRISMA-Children} OR {PRISMA-H}) |
| 7 | SLR/MA + Cochrane Handbook | Scopus  (25.04.2021) | SEARCH STRING #5 AND ALL({Cochrane Handbook} OR {Cochrane Collaboration Handbook} OR {Cochrane Collaboration Tool Kit} OR {Cochrane Reviewers’ Handbook})) |
| 8 | SLR/MA + PRISMA | Scopus  (25.04.2021) | SEARCH STRING #5 AND ALL({PRISMA statement} OR {PRISMA 2000 statement} OR {Preferred reporting items} OR {PRISMA-P} OR {PRISMA-E} OR {PRISMA-NMA} OR {PRISMA-Equity} OR {PRISMA-IPD} OR {PRISMA-C} OR {PRISMA-CI} OR {PRISMA-DTA} OR {PRISMA-ScR} OR {PRISMA flow} OR {PRISMA for Abstracts} OR {PRISMA-Children} OR {PRISMA-H})) |
| 9 | SLR/MA (FT only) | Lens.org (16.04.2021) | (title:("meta analysis") OR title:(“meta-analysis") OR title:("meta analyses") OR title:(“meta-analyses") OR title:(“MASEM") OR title:(“meta analytic") OR title:(“meta-analytic") OR title:(“meta-analytically") OR title:(“metaanalytically") OR title:(“meta-analytical") OR title:(“metaanalytical") OR title:(“metafor") OR title:(“metaSEM") OR title:(“meta-SEM") OR title:(“robumeta") OR title:(“meta regression") OR title:(“meta-regression") OR title:(“meta regressions") OR title:(“meta-regressions") OR title:(“metaregression") OR title:("metaanalysis") OR title:("metaanalyses") OR abstract:("meta analysis") OR abstract:(“meta-analysis") OR abstract:(“meta analyses") OR abstract:(“meta-analyses") OR abstract:(“MASEM") OR abstract:(“meta analytic") OR abstract:(“meta-analytic") OR abstract:(“meta-analytically") OR abstract:(“metaanalytically") OR abstract:(“meta-analytical") OR abstract:(“metaanalytical") OR abstract:(“metafor") OR abstract:(“metaSEM") OR abstract:(“meta-SEM") OR abstract:(“robumeta") OR abstract:(“meta regression") OR abstract:(“meta-regression") OR abstract:(“meta regressions") OR abstract:(“meta-regressions") OR abstract:(“metaregression") OR abstract:("metaanalysis") OR abstract:("metaanalyses")OR keyword:("meta analysis") OR keyword:(“meta-analysis") OR keyword:(“meta analyses") OR keyword:(“meta-analyses") OR keyword:(“MASEM") OR keyword:(“meta analytic") OR keyword:(“meta-analytic") OR keyword:(“meta-analytically") OR keyword:(“metaanalytically") OR keyword:(“meta-analytical") OR keyword:(“metaanalytical") OR keyword:(“metafor") OR keyword:(“metaSEM") OR keyword:(“meta-SEM") OR keyword:(“robumeta") OR keyword:(“meta regression") OR keyword:(“meta-regression") OR keyword:(“meta regressions") OR keyword:(“meta-regressions") OR keyword:(“metaregression") OR keyword:("metaanalysis") OR keyword:("metaanalyses")title:("systematic literature review") OR title:(“systematic review") OR title:(“systematic literature reviews") OR title:(“systematic reviews") OR title:(“systematically reviewed") OR title:(“Cochrane review") OR title:(“Cochrane systematic review") OR title:(“Cochrane reviews") OR title:(“Cochrane systematic reviews") OR title:(“Campbell review") OR title:(“Campbell systematic review") OR title:(“Campbell reviews") OR title:(“Campbell systematic reviews") OR title:(“PRISMA statement") OR title:("systematicreview") OR title:("systematicreviews") OR abstract:("systematic literature review") OR abstract:(“systematic review") OR abstract:(“systematic literature reviews") OR abstract:(“systematic reviews") OR abstract:(“systematically reviewed") OR abstract:(“Cochrane review") OR abstract:(“Cochrane systematic review") OR abstract:(“Cochrane reviews") OR abstract:(“Cochrane systematic reviews") OR abstract:(“Campbell review") OR abstract:(“Campbell systematic review") OR abstract:(“Campbell reviews") OR abstract:(“Campbell systematic reviews") OR abstract:(“PRISMA statement") OR abstract:("systematicreview") OR abstract:("systematicreviews") OR keyword:("systematic literature review") OR keyword:(“systematic review") OR keyword:(“systematic literature reviews") OR keyword:(“systematic reviews") OR keyword:(“systematically reviewed") OR keyword:(“Cochrane review") OR keyword:(“Cochrane systematic review") OR keyword:(“Cochrane reviews") OR keyword:(“Cochrane systematic reviews") OR keyword:(“Campbell review") OR keyword:(“Campbell systematic review") OR keyword:(“Campbell reviews") OR keyword:(“Campbell systematic reviews") OR keyword:(“PRISMA statement") OR keyword:("systematicreview") OR keyword:("systematicreviews")) |
| 10 | SLR/MA + Google Scholar (FT only) | Lens.org (16.04.2021) | SEARCH STRING #9 AND ("Google Scholar") NOT ("PubMed, CAS, Scopus and Google Scholar") |
| 11 | SLR/MA + SSS (FT only) | Lens.org (20.04.2021) | SEARCH STRING #9 AND ("Web of Science" OR "Web of Knowledge" OR "Scopus" OR "ProQuest" OR "PubMed" OR "Ovid" OR "Ebsco*" OR "cochrane central register of control trials" OR "Cochrane Library" OR "LILACS" OR "ACM Digital Library" OR "Bielefeld Academic Search Engine" OR "ClinicalTrials.gov" OR "ScienceDirect" OR "TRID" OR "Virtual Health Library" OR "Wiley Online") NOT ("PubMed, CAS, Scopus and Google Scholar") |
| 12 | SLR/MA + SSS + Google Scholar only (FT only) | Lens.org (20.04.2021) | SEARCH STRING #9 AND ("Web of Science" OR "Web of Knowledge" OR "Scopus" OR "ProQuest" OR "PubMed" OR "Ovid" OR "Ebsco*" OR "cochrane central register of control trials" OR "Cochrane Library" OR "LILACS" OR "ACM Digital Library" OR "Bielefeld Academic Search Engine" OR "ClinicalTrials.gov" OR "ScienceDirect" OR "TRID" OR "Virtual Health Library" OR "Wiley Online") AND ("Google Scholar") NOT ("PubMed, CAS, Scopus and Google Scholar") |

1. Gusenbauer M. Google Scholar to Overshadow Them All? Comparing the Sizes of 12 Academic Search Engines and Bibliographic Databases. Scientometrics. 2019;118(1):177-214. doi:10.1007/s11192-018-2958-5. [↑](#footnote-ref-1)
2. Delgado López-Cózar E, Orduna-Malea E, Martín-Martín A. Google Scholar as a data source for research assessment. In: Glaenzel W, Moed H, Schmoch U, eds. Springer Handbook of Science and Technology Indicators; 2018. [↑](#footnote-ref-2)
3. Jayabalasingham B, Boverhof R, Agnew K, Klein S. Identifying research supporting the United Nations Sustainable Development Goals. Mendeley Data, V1: Mendeley; 2019. [↑](#footnote-ref-3)
